# Functional assays of non-canonical splice-site variants in inherited retinal dystrophies genes

**DOI:** 10.1038/s41598-021-03925-1

**Published:** 2022-01-07

**Authors:** Ana Rodriguez-Muñoz, Alessandro Liquori, Belén García-Bohorquez, Teresa Jaijo, Elena Aller, José M. Millán, Gema García-García

**Affiliations:** 1 Molecular, Cellular and Genomics Biomedicine Research Group, Instituto de Investigación Sanitaria La Fe (IIS La Fe), Valencia, Spain; 2 Unidad Mixta de Enfermedades Raras IIS La Fe-Centro de Investigación Príncipe Felipe, Valencia, Spain; 3 Hematology Research Group, Instituto de Investigación Sanitaria La Fe, Valencia, Spain; 4 Centro de Investigación Biomédica en Red de Cáncer (CIBERONC), Madrid, Spain; 5 Biomedical Research Network for Rare Diseases, CIBERER, Madrid, Spain; 6 Genetics Unit, Hospital Universitario y Politécnico La Fe, Valencia, Spain

**Keywords:** Genetics, Molecular biology, Molecular medicine

## Abstract

Inherited retinal dystrophies are a group of disorders characterized by the progressive degeneration of photoreceptors leading to loss of the visual function and eventually to legal blindness. Although next generation sequencing (NGS) has revolutionized the molecular diagnosis of these diseases, the pathogenicity of some mutations casts doubts. After the screening of 208 patients with a panel of 117 genes, we obtained 383 variants that were analysed in silico with bioinformatic prediction programs. Based on the results of these tools, we selected 15 variants for their functional assessment. Therefore, we carried out minigene assays to unveil whether they could affect the splicing of the corresponding gene. As a whole, seven variants were found to induce aberrant splicing in the following genes: *BEST1*, *CACNA2D4, PRCD, RIMS1, FSCN2, MERTK* and *MAK*. This study shows the efficacy of a workflow, based on the association of the Minimum Allele Frequency, family co-segregation, in silico predictions and in vitro assays to determine the effect of potential splice site variants identified by DNA-based NGS. These findings improve the molecular diagnosis of inherited retinal dystrophies and will allow some patients to benefit from the upcoming gene-based therapeutic strategies.

## Introduction

Inherited retinal dystrophies (IRD) encompass a clinically and genetically heterogeneous group of disorders characterized by the progressive degeneration of rod and cone photoreceptors, leading to loss of the visual function and legal blindness. Clinical hallmarks of IRD include blurry vision, photophobia, colour vision abnormalities, visual field loss and nyctalopia^1^. To date, 271 genes related to IRD have been identified (https://sph.uth.edu/retnet/sum-dis.htm#A-genes, accessed August 2021).

The identification of the disease-causing mutations is crucial for patient genetic counselling and to benefit from clinical trials or new treatments. The next-generation sequencing (NGS) has revolutionized the molecular diagnosis of these genetically heterogeneous diseases and the percentage of genetically diagnosed patients has increased dramatically^2–4^. On the other hand, NGS unveils a high number of variants per patient, many of which have a low allele frequency in the population and, therefore, could be involved in rare diseases. In silico predictions are crucial to evaluate the pathogenic effects of these variants, including missense, silent and putative splicing changes. Nevertheless, in most of the cases, the clinical significance of them remains unknown.

In agreement with the American College of Medical Genetics and Genomics (ACMG) standards, functional studies are a powerful tool in support of pathogenicity assessment^5^. In contrast to protein function assays, characterization of potential splicing variants is more feasible. However, most of the genes involved in IRD are only expressed in non-accessible tissues and/or mRNA from patients is often unavailable. In these cases, in vitro minigene splicing assays are a good alternative to assess the impact of variants at the mRNA level^6^.

In recent years it has been shown that the use of antisense oligonucleotides (AONs) is a promising therapeutic approach to correct aberrant splicing. AONs are short single stranded molecules designed to target and modulate (pre-)mRNA splicing. AONs can be designed to bind directly splice sites or cis-regulatory elements in order to produce an exon-skipping or specifically mask altered splice-sites involved in aberrant splicing. This strategy has been shown to be useful as a therapy for different diseases, including retinal dystrophies^7–10^. Of note, AONs has been used to correct the aberrant pre-mRNA splicing produced by deep-intronic mutations in *USH2A* or *CEP290*^10–13^.

In our cohort of 208 IRD patients, we identified 15 putative non-canonical splicing mutations as a result of the sequencing of 117 genes. The aim of this study was to predict the pathogenic nature of these variants and to assess their effect on the splicing process by minigene assays.

## Results

### Genetic variants

Once variants with a MAF > 0.01 were discarded, 383 intronic and exonic variants identified in the IRD genes were studied with bioinformatic tools (HSF and MaxEnt) to analyze their effect on splicing. Variants were selected for further functional assays according to the criteria mentioned in Material and Methods section and independently from their involvement on the phenotype of the individual (i.e., any variant found in a IRD gene with a putative splicing effect). Table 1 shows the results obtained for the HSF, MaxEnt and SpliceAI predictors for each of the selected variants.

**Table 1:** Results of the prediction programmes for the selected variants.

Gene	Mutation	SS	Position	HSF			MaxEnt			SpliceAI^a^
				Wt	Mut	Variation (%)	Wt	Mut	Variation (%)	
*ABCA4*	c.2972G>T	D	E, 54/132	64.62	91.45	+ 41.52	0.41	8.05	+ 2063.41	DG: 0.09 (2)
*ABCA4*	c.6148G>C	A	E, 1/135	87.36	83.20	− 4.76	12.46	9.41	− 24.48	AL: 0.00 (0)
***BEST1***	c.637_639del	A	E, 1_3/78	93.39	91.03	− 2.53	–	–	–	AL: 1.00 (3)
				75.49	91.03	+ 20.59	11.00	11.68	+ 6.18	AG: 0.93 (6)
***CACNA2D4***	c.2153-12_2155del	A	I, − 12_− 1	93.15	57.22	− 38.57	8.53	− 2.70	− 131.65	–
			E, 1_3/94							
*CNNM4*	c.2039C>T	D	E, 91/182	55.92	82.75	+ 47.98	1.89	5.86	+ 409.52	DG: 0.01 (− 2)
***FSCN2***	c.1105G>A	D	E, 122/122	–	76.95	–	–	9.04	–	DG: 0.80 (20)
				89.94	79.36	− 11.76	9.10	4.04	− 55.60	DL:0.47 (0)
*IDH3B*	c.532A>G	A	E, 1/134	88.49	91.62	+ 3.54	6.89	7.89	+ 14.51	AG: 0.01 (− 8)
***MAK***	c.755A>G	A	E, 92/168	59.74	88.69	+ 48.46	− 3.58	5.16	+ 244.13	AG: 0.68 (− 1)
				81.74	81.69	− 0.06	3.75	− 2.2	− 158.67	AL: 0.34 (− 7)
***MERTK***	c.1450G>A	D	E, 154/154	–	82.74	–	–	5.74	–	DG: 0.43 (16)
				–	80.63	–	–	3.44	–	
				90.80	80.22	− 11.65	8.94	3.45	− 61.41	DL: 0.67 (0)
***PRCD***	c.74 + 5G>C	D	I, + 5	79.79	67.78	− 15.05	4.27	− 3.52	− 182.44	DL: 0.96 (− 5)
*PRPF8*	c.434 + 3G>A	D	I, + 3	85.58	86.23	+ 0.76	4.74	7.83	+ 65.19	DG: 0.00 (3)
*PRPF31*	c.182C>G	D	E, 5/61	62.94	89.77	+ 42.63	− 1.98	6.29	+ 417.68	DG: 0.00 (− 1)
*RHO*	c.316G>A	A	E, 316/361	64.15	93.10	+ 45.13	− 1.61	6.34	+ 493.79	AG: 0.00 (2)
***RIMS1***	c.2544 + 4A>G	D	I, + 4	82.83	74.49	− 10.07	4.44	− 1.23	− 127.70	DL: 0.29 (− 4)
*RPGRIP1*	c.930 + 3A>G	D	I, + 3	96.23	85.39	− 11.26	8.40	4.72	− 43.81	–

*A* acceptor, *AG* acceptor gain, *AL* acceptor loss, *D* donor, *DG* donor gain, *DL* donor loss, *E* exon, *I* intron, *Mut* mutant, *SS* splice-site, *Wt* wildtype, *ABCA4*: NM_000350.2*, BEST1*: NM_004183.4, *CACNA2D4*: NM_172364.4, *CNNM4*: NM_020184.3, *FSCN2*: NM_012418.4, *IDH3B*: NM_001258384.2, *MAK*: NM_005906.5, *MERTK*: NM_006343.2, *PRCD*: NM_001077620.3, *PRPF8*: NM_006445.4, *PRPF31*: NM_015629.4, *RHO*: NM_000539.3, *RIMS1*: NM_014989.6, *RPGRIP1*: NM_020366.4

^a^Distance in bp.

–Gene not available in the consulted website.

### Minigene assays

**Figure 1:**
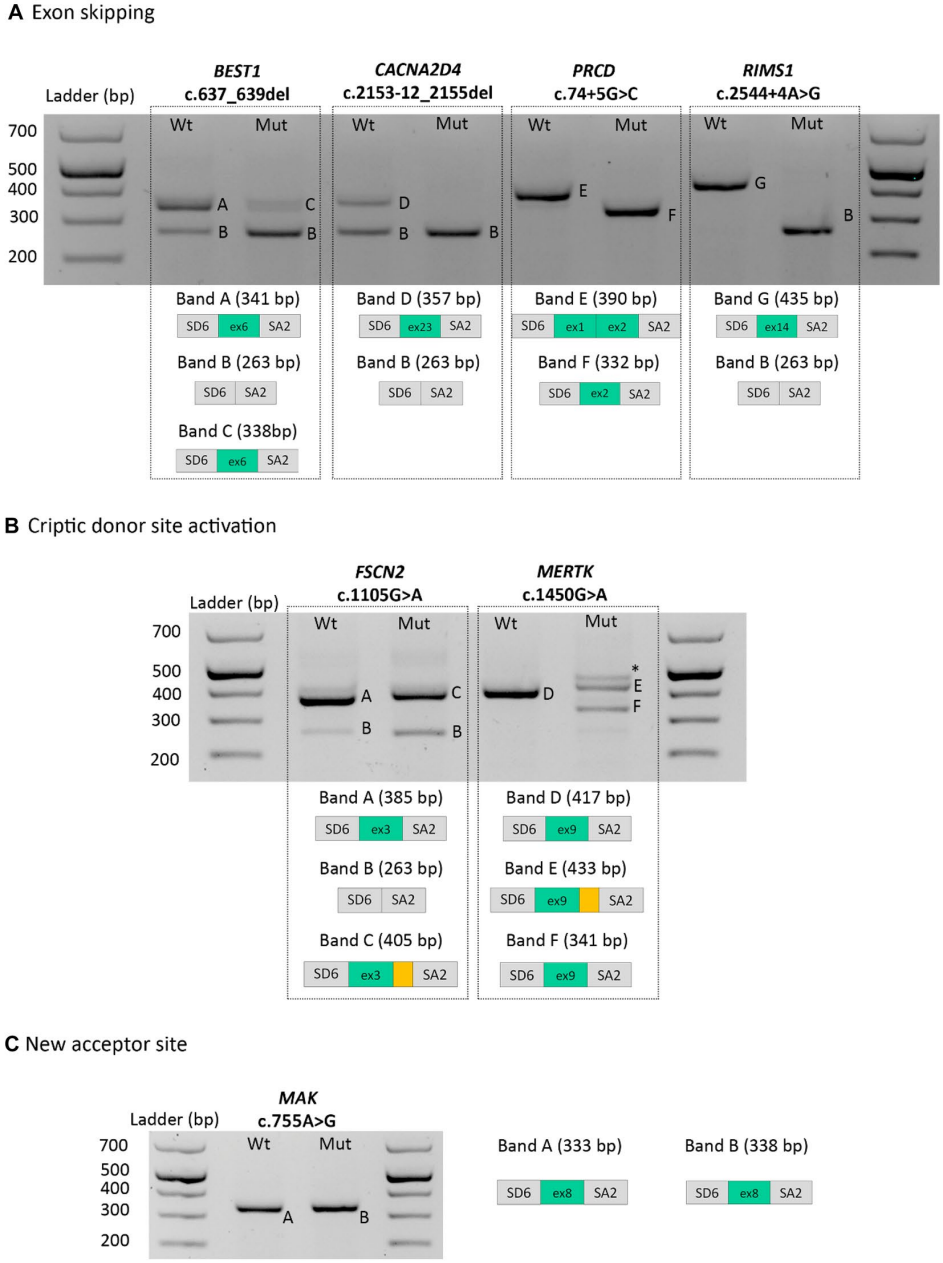
Representation of the amplified products are depicted below the agarose gel. Grey boxes represent pSPL3 resident exons SD6 and SA2 and green boxes exons from the respective genes mutated. Yellow box represents retained intronic sequence. The asterisk indicates band considered as heteroduplex formation.

Minigene assays were performed on 15 variants found in 15 patients, respectively. Results of this analysis are presented in Fig. 1 and Supplementary Fig. 1. Overall, seven variants were found to have an aberrant splicing compared with wildtype minigene: four by the loss of the natural acceptor or donor splice site (*BEST1* c.637_639del, *CACNA2D4* c.2153-12_2155del, *PRCD* c.74 + 5G>C, *RIMS1* c.2544 + 4A>G, Fig. 1A); two by the activation of a cryptic donor splice site (*FSCN2* c.1105G>A, *MERTK* c.1450G>A, Fig. 1B); and one by the creation of a new acceptor splice site (*MAK* c.755A>G, Fig. 1C). Normal splicing patterns were observed in mutated minigenes of the remaining eight cases (Supplementary Fig. 1).

Among the four variants altering the acceptor splice sites (3′ss) recognition (Table 1), two were deletions (*BEST1* c.637_639del, *CACNA2D4* c.2153-12_2155del), and two substitutions occur in the first position of the exon (*ABCA4* c.6148G>C, *IDH3B* c.532A>G).

The *BEST1* c.637_639del mutation involves the deletion of the first three nucleotides of exon 6. The results of the SpliceAI predictor for this mutation indicate that two events would occur: the loss of the natural acceptor splice site and the activation of a cryptic acceptor splice site. These predictions were confirmed by minigene findings. In contrast, the HSF and MaxEnt algorithms did not correctly predict these two events. Taking into account the result of minigene assay. this deletion in exon 6 would not alter the reading pattern, likely resulting in the mutated protein p.(Glu212_Gln238del). However, the mutated construct revealed a major aberrant transcript corresponding to the exon 6 skipping (band B) and a faint product corresponding to the deletion of the first three bp of the exon 6, as a consequence of the cryptic acceptor site activation (Fig. 1A, band C). Therefore, if the major aberrant transcript was translated, it would encode for the p.(Glu212_Gln238del) protein, with a loss of 26 amino acids with respect to the wildtype protein. According to the NCBI Conserved Domains predictor, the bestrophin domain would not disappear in the mutated protein. However, according to the dbPTM database, phosphorylation at the residue 227 of the BEST1 protein would be lost.

In the case of the c.2153-12_2155del (*CACNA2D4*) variant, the mutated construct generated an aberrant transcript corresponding to the skipping of exon 23 (Fig. 1A, band B). The mutation deleted the natural acceptor site. This would result in the loss of the reading pattern and could lead to mRNA degradation mediated by terminator mutations (known as nonsense-mediated mRNA decay, NMD), as supported by the predictor NMDEsc. If NMD would not occur, the mutated protein p.(Cys718Trpfs*19) would be produced, without the VGCC-alpha2 domain.

No splicing effect was observed for *ABCA4* c.6148G>C and *IDH3B* c.532A>G.

When variants altering donor splice sites (5′ss) were considered, the six studied variants consisted of four intronic changes, localized at position + 3 (*PRPF8* c.434 + 3G>A, *RPGRIP1* c.930 + 3A>G), + 4 (*RIMS1* c.2544 + 4A>G) and + 5 (*PRCD* c.74 + 5G>C); and two variants localized at the last position of an exon, affecting the G consensus nucleotide at the position − 1 of 5′ss motifs (*FSCN2* c.1105G>A, *MERTK* c.1450G>A) (Table 1).

Among the intronic variants, only the mutations c.2544 + 4A>G in *RIMS1* and c.74 + 5G>C in *PRCD* induced aberrant splicing in minigenes assays, leading to the complete skipping of the closest exon.

Mutant minigene of c.74 + 5G>C (*PRCD*) variant showed one transcript corresponding to the skipping of exon 1 (Fig. 1A, band F). This mutation avoided the recognition of the natural donor splice site leading to a deletion of the first exon of *PRCD*.

The mutated construct of the c.2544 + 4A>G (*RIMS1*) variant generated an aberrant transcript corresponding to an exon skipping (Fig. 1A, band B). Skipping of exon 14 of *RIMS1* would result in the loss of the reading pattern and NMD-mediated mRNA degradation, as predicted by the NMDEsc tool. If NMD would not occur, the mutated protein p.(Ser791Argfs*4) would be produced without the highly conserved domains C2A_RIM1alpha and C2B_RIM1alpha.

As expected from the in silico predictors, both substitutions located at exonic position − 1 induced the loss of the natural 5′ss and the activation of different cryptic donor splice sites.

The *FSCN2* c.1105G>A variant led to the exclusive use of an intronic cryptic 5′ss (HSF: 76.95, MaxEnt: 9.04), leading to the out-of-frame inclusion of the first 20 nucleotides of *FSCN2* intron 3 in the mature transcript. The processing of the mutated minigene generated two aberrant transcripts as shown in Fig. 1B: band C, in which an insertion of 20 intronic bp was observed, corresponding to donor cryptic site activation CAGgtactg (HSF: 76.95, MaxEnt: 9.04), and band B, corresponding to the loss of exon 3. The new mutated r.1105_1106ins[1105 + 1_1105 + 20] transcript (band C), according to the NMDEsc predictor, would not be degraded and would give rise to the p.(Lys370Glufs*50) protein, lacking one of the four fascin domains. On the other hand, the transcript r.984_1106del would be susceptible to degradation according to the NMDEsc predictor. If NMD would not occur, the mutated protein p.(Ser329Glnfs*84) would be produced without two conserved fascin domains.

The *MERTK* c.1450G>A mutation activated, on the one hand, a downstream cryptic 5′ss (HSF: 82.74, MaxEnt: 5.74), leading to the out-of-frame inclusion of the first 16 bp of *MERTK* intron 9, and, on the other hand, an exonic cryptic 5′ss (HSF: 80.63, MaxEnt: 3.44), leading to the loss of the last 76 nucleotides of *MERTK* exon 9*.* Mutant minigene showed two aberrant transcripts (Fig. 1B): band E, in which an insertion of 16 intronic bp was observed, as result of the donor cryptic site activation CCAgtaagg (HSF: 82.74, MaxEnt: 5.74), and band F, in which a deletion of 76 bp in the 5′ end of exon 9 was observed, corresponding to the donor cryptic site activation ACAgtgagg (HSF: 80.63, MaxEnt: 3.44). Both transcript r.1450_1451ins[1450 + 1_1450 + 16] and transcript r.1374_1450del, would be susceptible to degradation, according to the NMDEsc predictor. If NMD would not occur, the mutated proteins p.(Trp485Glufs*63) and p.(Val459Leufs*60), respectively, would be produced. In both cases the protein tyrosine kinase domain (588–852 aa) would be absent.

Exonic mutations that were distant both from the 5′ and 3′ss could activate or create a new splice site and result in splicing defects. We studied three variants whose in silico results predict a new donor site (*ABCA4* c.2972G>T, *CNNM4* c.2039C>T, *PRPF31* c.182C>G) and two new acceptor site (*MAK* c.755A>G, *RHO* c.316G>A) (Table 1). According with the SpliceAI predictor, only the *MAK* c.755A>G variation showed defects in splicing, that creates a new acceptor splice site 92 nucleotides downstream of the exon junction (Fig. 1C). Although, exon 8 canonical acceptor splice site of this gene was not recognized in both wild-type and mutated constructions, missplicing of mutated minigene should be interpreted from the comparison with the wildtype construct. Indeed, a cryptic acceptor splice site in c.760-bp position was activated in the case of wild-type minigene instead of the natural one. However, this was different to acceptor splice observed in mutated construction, where a novel site was activated as a result of the c.755A>G change and according to in silico analysis. Therefore, a 5-bp difference was found between mutated and wild-type minigenes and this difference might increase to 92-bp when distance between aberrant and canonical acceptor splice sites is considered (Fig. 1C, band B). The mutation generated a new acceptor site tcttattcccagTG (HSF: 88.69, MaxEnt: 5.16). The aberrant r.664_755del transcript, according to the NMDEsc predictor, would be susceptible to degradation. If NMD would not occur, it would give rise to the mutated protein p.(Ser222Cysfs*3), with the protein kinase domain (4–284 aa) truncated.

## Discussion

Mutations affecting pre-mRNA processing are not insignificant in the population. To date, an 8.7% out of the total number of variants published in HGMD Professional 2020.4 (http://www.hgmd.cf.ac.uk/ac/index.php, accessed April, 2021) (26,610/306,768), are disease-causing mutations affecting splicing. However, this estimation still seems conservative because mutations in coding regions are usually considered as missense, nonsense, or silent, which may lead to misclassification of splicing mutations and underestimation of their number^14^.

It is difficult to predict the consequences of missense, synonymous and intronic variants, to discriminate neutral variants from those with pathogenic effect. Variants that putatively affect the splicing mechanism are usually considered pathogenic based on the conservation of their canonical splicing site, their absence in control population, co-segregation with the disease in families and the results of bioinformatics predictors^15^. In this work, HSF and MaxEnt algorithms have been used to predict the effect on splicing of the variants identified in our cohort. The interpretation of the in silico data and the establishment of thresholds were chosen based on previous work^16^. Following these criteria, 15 changes were selected that could potentially affect this mechanism and were further studied with the SpliceAI predictor (Table 1). Of note, of the three in silico predictors used, SpliceAI was the only one that predicted all the results observed in the in vitro assays. However, these results are only computational predictions, so according to the ACMG criteria, functional studies are required to confirm the effect on mRNA processing. In vitro assays using minigenes have been found to be a useful approach to determine the effect of these variants when genes have a restricted expression profile and the specific tissue sample is difficult to obtain^17^.

In contrast to variants affecting the canonical splice sites, which in approximately two-thirds of cases lead to exon skipping, the effect of NCSS variants can vary greatly depending on the sequence alteration and its environment^18^. It is acknowledged that not all the positions of 5′ss motif contribute equally to the basepairing with the splicing factors. Excluding the invariant + 1 and + 2 positions, the positions − 1 and + 5 would be the most important^18,19^, as shown also in our minigene assays.

Bestrophin-1 is a channel protein that conducts anions across the EPR membrane^20^. Alteration of the primary sequence of this protein would have a negative impact on ion conductance, consistent with the decreased or absent response in the EOG described in patients with Best's disease^21^. Histopathological studies of the retina of a patient with Best's disease and the p.(Tyr227Asn) mutation suggest that this mutation could result in erroneous transport of bestrophin-1 to its destination in the cell^22^. Subsequently, Doumanov et al. demonstrated that the Tyr227AspTrpIle motif is essential in the proper delivery of bestrophin-1 to the basolateral membrane of EPR cells^23^. Therefore, we consider that exon 6 of *BEST1* is essential in the function of the protein and the c.637_639del variant would be potentially pathogenic.

The *ABCA4* c.6148G>C and *IDH3B* c.532A>G variants, although they did not meet the impact threshold established for the predictors, were studied in vitro because they were located in the first position of the exon. As expected, none of them was shown to alter the correct processing of the pre-mRNA, in agreement with the in silico results. However, the *ABCA4* c.6148G>C was studied because there is no consensus on the clinical interpretation of this variant in the literature. This was first described by Allikmets et al. as c.6034G>C p.(Val2012Leu), identified in heterozygosity, in two independent families diagnosed with STGD^24^, and has traditionally been considered as a pathology-causing mutation^2,25^. However, an exhaustive literature review by Cornelis et al. classifies this variant of uncertain clinical significance^26^. Furthermore, in many of the cases in which the p.(Val2050Leu) variant has been identified, the second mutation has not been identified and/or has been identified in resolved patients with mutations in other genes and RP phenotype^27–30^. For all these reasons, we believe that this variant should be interpreted with caution, especially in the context of genetic and reproductive counselling.

In this study we found a variant in *FSCN2* that potentially alters the splicing of the gene likely leading to a truncated protein. The encoded protein Fascin2 is an actin cross-linking protein that is mainly expressed in the photoreceptors^31,32^. *FSCN2* was firstly associated to autosomal dominant retinitis pigmentosa (RP) in the Japanese population^33^. However, truncating mutations that did not co-segregate with the disease in a Spanish family and in a Chinese cohort cast doubts about the real involvement of *FSCN2* in RP^34,35^.

Based on the literature, the majority of NCSS variants studies in this work (n = 4, 60%) affected the splice donor site. According to Sangermano et al., the longer intronic consensus of the splice acceptor sites (− 14 to + 1) in which the pyrimidine to pyrimidine substitutions have virtually low effect on the predicted splice strengths, may explain why variants that affect the splice acceptor site are generally less perturbative^14^.

In agreement with the results obtained in this work, independently from their type, all the variants suspected to be pathological should be analyzed by bioinformatics tools, and when high score variations are observed, such variants should be studied by in vitro assays, to avoid both false positive and false negative results.

The splicing events observed by minigenes do not always reflect the actual process in the affected tissues. The size restriction of the constructs represents a well-known limitation to fully assess the effect of variants in non-canonical splicing sites. In addition, in a few cases this issue can also affect the recognition of canonical splice sites; such as in MAK minigenes where the chimeric genomic context created between pSPL3 vector and insert sequences led to the recognition of a cryptic splice site instead of the canonical one. In other cases (e.g., *FSCN2* minigenes), a faint transcript with a canonical skipped exon is observed in wildtype minigene. Therefore, as shown here, it is important to study variants at non-canonical splicing sites in the context of their upstream or downstream introns and exons, as their effect is often influenced by other intronic cis-regulatory elements or affects exons located at considerable distances. Another drawback of the in vitro splicing assay is that HEK293 cells do not always mimic splicing defects that might occur in the patient's retina. Indeed, there are studies indicating that specific splicing factors are present in the retina^36,37^. Despite this, minigenes are a good approach to determine the pathogenic nature of splicing variants when it is difficult to obtain RNA from patient tissue, as in the case of IRD genes, which most of them present a restricted expression profile associated with photoreceptors and RPE cells.

Interestingly, this study has allowed to reclassify, as mutations affecting splicing, three mutations previously categorized as missense: *FSCN2* c.1105G>A, *MAK* c.755A>G and *MERTK* c.1450G>A; and two deletions: *BEST1* c.637_639del and *CACNA2D4* c.2153-12_2155del. Determining the disease mechanism of splice-site variants will provide new opportunities for therapeutic approaches for patients carrying these variants. Antisense oligonucleotide-mediated exon-skipping is a hopeful therapeutic approach, as encouraging results were obtained in the already completed phase I/II clinical trials (https://clinicaltrials.gov/ct2/show/NCT03140969) for the most frequent deep-intronic mutation in *CEP290* that cause Leber congenital amaurosis^12,13^.

This study demonstrates the efficacy of an integrated approach, constituted by MAF, co-segregation, in silico predictions and in vitro assays to determine the effect of potential splice site variants identified by high-throughput DNA sequencing. In this report, we have studied 15 different changes identified in IRD genes by the minigene strategy, confirming the pathogenicity of seven of them. Predictions by splice-site algorithms are crucial to evaluate the pathogenic effects of putative splicing variants and for the clinical interpretation of previously misclassified missense and splice variants. These findings improve the diagnosis of IRD patients who could benefit from the upcoming gene-based therapeutic strategies.

## Materials and methods

### Patients and molecular diagnosis

Genomic DNA was isolated from 208 patients diagnosed with non-syndromic inherited retinal dystrophies (NS-IRD) from 124 unrelated pedigrees. Written informed consent was obtained from all participants or their legal guardians. This study was approved by the Hospital La Fe Ethics Committee in agreement with the Declaration of Helsinki.

Genetic testing, data analysis and variant interpretation were performed as described previously^4^. Briefly, NGS analysis was performed by target enrichment with a custom panel of 117 IRD-associated genes (Agilent Technologies, Santa Clara, CA, USA) and libraries were sequenced on an Illumina MiSeq platform (Illumina, San Diego, CA, USA). Data analysis was performed using SureCall software (Agilent Technologies, Santa Clara, CA, USA) and variant annotation employing wANNOVAR. Variants were classified according to standards of the American College of Medical Genetics and Genomics (ACMG)^5^.

### In silico analysis and selection of variants

To evaluate the effect of the identified variants in the splicing process and recognition of donor and acceptor sites, in silico studies were first performed. Two different algorithms: Human Splicing Finder (HSF) and MaxEnt, included in the splicing predictor tool HSF 3.1 (http://www.umd.be/HSF/index.html)^38^ were used for this purpose. Candidate variants for the functional assay were selected according to the cut-offs defined by Desmet et al.^38^: 5′ or 3′ splice sites scores > 3 for MaxEnt and > 65 for HSF were combined with a 30% and 10% score variation between wild-type and mutated sequences, respectively. In addition, mutations at position + 4 and at position + 3 or + 5 with a score variation of 7% and 14%, respectively, were included. Furthermore, variants located in the first position of the exon and matching one or none of the above criteria were selected: *ABCA4*: c.6148G>C, *BEST1*: c.637_639del and *IDH3B*: c.532A>G. Mutations located at canonical splicing sites were not selected for the minigene assay. The mutations selected for the in vitro assay were also analyzed using the new generation predictor SpliceAI^39^ included in the MobiDetails platform (https://mobidetails.iurc.montp.inserm.fr/MD/). Variants were analysed independently from their putative association with the phenotype of the patient. Clinical diagnosis and genotype of patients carrying the variants selected for the minigene assay is detailed in Supplementary Table 1. Co-segregation analysis was performed when DNA from family member was available (Supplementary Fig. 2).

### Minigene assay

Minigenes were used to assess the impact of selected variants on the mRNA processing.

For all the selected variants, involved exons and their flanking intronic sequences (~ ± 200 bp for each side) were amplified from the patients’ DNA using the High Fidelity Phusion polymerase (Thermo Fisher Scientific Waltham, MA, USA) with specific primers containing an additional XhoI and NheI restriction site (Supplementary Table 2). Amplicons were inserted between the XhoI and NheI restriction sites using T4 DNA ligase (Thermo Fisher Scientific) into the pSPL3 exon-trapping vector (kindly provided by Dr. I. Botillo and Dr. S. Tuffery-Giraud). *Escherichia coli* competent cells (XL1-Blue Supercompetent Cells, Agilent Technologies, Santa Clara, CA, USA) were transformed with the plasmid construction by heat shock. pSPL3 vector has not been developed for the study variants within first and last exons, since these are preceded and followed by UTRs, respectively. Therefore, to facilitate the recognition of *PRCD* exon 1 by spliceosome, we modified by directed mutagenesis (QuikChange II XL Site-Directed Mutagenesis Kit) a decoy acceptor splice site identified in silico within this exon obtaining a stronger acceptor splice site, as endorsed by in silico and functional analyses (i.e. wild-type minigene). To this aim, we modified 4 positions (as shown in the figure) in order to increase the acceptor site prediction score from 88.43 to 96.30 for HSF and 7.39–12.6 for MaxEnt (Supplementary Table 3 and Supplementary Fig. 3). The sequence of each minigene construction was verified by direct sequencing.

Subsequently, one microgram of each wild-type and mutated construct were transfected with the Lipofectamine™ 3000 reagents (Thermo Fisher Scientific) into HEK293 cells grown in six-well plates, according to manufacturer’s instructions. All the transfection experiments in HEK293 cells were performed in duplicate. Twenty-four hours after transfection, cells were harvested and total RNA was isolated by the RNeasy Mini kit (Qiagen, Hilden, Germany). Then, cDNA synthesis was carried out using the PrimeScript RT Reagent Kit (TaKaRa, Kusatsu, Japan) according to the manufacturer’s recommendations. The cDNA was amplified using the pSPL3-specific SD6 forward and SA2 reverse primers^34^. PCR products were visualized on a 1% agarose gel and bands were purified with the QIAquick Gel Extraction Kit (Qiagen, Hilden, Germany) and directly sequenced in both senses by Sanger method (BigDye Terminator kit v3.1, Applied Biosystems).

### Interpretation of minigenes assay

We used the Name Checker tool of Mutalyzer 2.032 (https://www.mutalyzer.nl/name-checker) for the annotation of the transcripts resulting from the minigene assays together with their protein products. The NMDEscPredictor bioinfomatics predictor (https://nmdprediction.shinyapps.io/nmdescpredictor/) was also used to test whether the newly mutated transcript could be degraded by the Nonsense Mediated Decay (NMD) pathway. In addition, the domains of the affected proteins were consulted in the NCBI Conserved domains database (https://www.ncbi.nlm.nih.gov/Structure/cdd/wrpsb.cgi?) and the possible post-transcriptional modifications of the protein in the cBioPortal database (https://www.cbioportal.org/).

## Supplementary Information

Supplementary Information.
