# Supplementary material for: Functional assays of non-canonical splice-site variants in inherited retinal dystrophies genes
Source: Sci Rep. 2022 Jan 7;12:68. doi: 10.1038/s41598-021-03925-1 (PMC8742059; doi:10.1038/s41598-021-03925-1)
Supplement: Supplementary file 1 — Supplementary Information. [file 41598_2021_3925_MOESM1_ESM.pdf]

Supplementary Information File

Functional assays of non-canonical splice-site variants in inherited retinal dystrophies genes.

Ana Rodriguez-Muñoz[&1,2], Alessandro Liquori[&3,4], Belén García-Bohorquez[1,2,5], Teresa Jaijo[1,2,5,6], Elena Aller[1,2,5,6], José M. Millán*[† 1,2,5], Gema García-García[†1,2,5]

Supplementary Table 1. Detailed information about the clinical and genetic diagnosis from patients carrying putative splicing-affecting variants.

Patient ID	Phenotype	Gene:transcript	Coding DNA	Protein	State	Classification*
RPN-410[#]	STGD	***ABCA4*:NM_000350**	**c.G2972T**	**p.G991V**	**het**	**LP**
		ABCA4:NM_000350	c.4253+4C>T	p.?	het	P
RPN-536	RP	***ABCA4*:NM_000350**	**c.G6148C**	**p.V2050L**	**het**	**US**
		CNGA1:NM_001142564	c.C1892T	p.T631M	het	LP
		CNGB1:NM_001297	c.3150delG	p.(Phe1051Leufs*12)	het	P
RPN-106[#]	BMD	*BEST1*:NM_004183	c.T602C	p.I201T	het	LP
		***BEST1*:NM_004183**	**c.637-2_637del**	**p.Glu213del**	**het**	**LP**
RP-677	STGD	***CNNM4*:NM_020184**	**c.C2039T**	**p.A680V**	**het**	**US**
RPN-304[#]	MD STGD-like	***FSCN2*:NM_012418**	**c.G1105A**	**p.G369S**	**het**	**US**
		PRPH2:NM_000322	c.G499A	p.G167S	het	P
RPN-299	RP	*C21orf2*:NM_004928	c.G172T	p.V58L	het	US
		***IDH3B*:NM_001258384**	**c.A532G**	**p.S178G**	**het**	**US**
RPN-326	BMD	*ABCA4*:NM_000350	c.C5642T	p.A1881V	het	LP
		BEST1:NM_004183	c.C698A	p.P233Q	het	LP
		***MAK*:NM_005906**	**c.A755G**	**p.N252S**	**het**	**US**
RPN-442[#]	RP	*MERTK*:NM_006343	MERTK del (chr2:111371701-113132395)		het	P
		***MERTK*:NM_006343**	**c.G1450A**	**p.G484S**	**het**	**LP**
RPN-451[#]	RP	*PRCD*:NM_001077620	c.102_111dup	p.Ser38X	het	P
		***PRCD*:NM_001077620**	**c.74+5G>C**	**p.?**	**het**	**LP**
RPN-461[#]	RP	***PRPF8*:NM_006445**	**c.434+3G>A**	**p.?**	**het**	**US**
		ROM1:NM_000327	ROM1 dup	p.?	het	LP
RPN-284	STGD	***PRPF31*:NM_015629**	**c.C182G**	**p.A61G**	**het**	**US**
RPN-253[#]	RP	***RHO*:NM_000539**	**c.G316A**	**p.G106R**	**het**	**P**
RPN-104[#]	LCA	*AIPL1*:NM_014336	c.97_104dup	p.(Phe35Leufs*2)	hom	P
		IQCB1:NM_001023570	c.1518_1519del	p.(His506Glnfs*13)	het	P
		***RIMS1*:NM_014989**	**c.2544+4A>G**	**p.?**	**het**	**US**
RP-632	RP	*EYS*:NM_001142800	ex. 32-33 del	p.?	het	P
		***RPGRIP1*:NM_020366**	**c.930+3A>G**	**p.?**	**het**	**US**
RPN-424	RD	***CACNA2D4*: *NM_172364.5***	**c.2153-12_2155del**	**p.?**	**het**	**US**

In bold are marked the variants analyzed by minigene assay. [#] Cases with segregation analysis carried out. *Classification prior to the minigenes analysis. Abbreviations: STGD: Stargardt disease; RP: retinitis pigmentosa; BMD: Best macular dystrophy; MD STGD-like: autosomal dominant macular dystrophy STGD disease-like; LCA: Leber congenital amaurosis; RD: Retinal dystrophy; P: Pathogenic. LP: Likely pathogenic. US: Uncertain significance.

Supplementary Table 2. Primers used to amplify the specific insert.

Primer	Sequence (5'-3')	Size (bp)	Flanking Intronic Region (Upstream / Downstream bp)
ABCA4(20D)_XhoI<br>ABCA4(20R)_NheI	**AAGAAT***CTCGAG*GGGAGCACCATGAAACAAGT<br>**AAGAAT***GCTAGC*GGAGGAGCCCTCAGCTCT	593	225 / 218
ABCA4(45D)_XhoI<br>ABCA4(45R)_NheI	**AAGAAT***CTCGAG*CGAGACCCACTGTCTTGCTC<br>**AAGAAT***GCTAGC*ACAGGAGACATCCTGGGAAC	676	290 / 248
BEST(6D)_XhoI<br>BEST(6R)_NheI	**AAGAAT***CTCGAG*CTGAGGGTCTTCCGAGAGC<br>**AAGAAT***GCTAGC*TCAGCTTCCCAAAGTGCTG	654	296 / 261
CACNA2D4 (23D)_XhoI<br>CACNA2D4 (23R)_NheI	**AAGAAT***CTCGAG*TGAGGTCACTGAGGGTCTCC<br>**AAGAAT***GCTAGC*CAACCCAGAGAAAGGAGCTG	566	268 / 204
CNNM4(6 D)_XhoI<br>CNNM4(6 R)_NheI	**AAGAAT***CTCGAG*TGTTTGGTTGATTTGTTTTGG<br>**AAGAAT***GCTAGC*AGCCACATGCACACCTGTAG	688	251 / 255
FSCN2 (3D)_XhoI<br>FSCN2 (3R)_NheI	**AAGAAT***CTCGAG*CATCACCTGCACCATCACTC<br>**AAGAAT***GCTAGC*TCCCAGCAGCGGAGATAAG	557	233 / 202
IDH3B(7D)_XhoI<br>IDH3B(7R)_NheI	**AAGAAT***CTCGAG*ATATGCGGCTGAGGTAGGTG<br>**AAGAAT***GCTAGC*TCCCATAGAGATTGGGCATC	800	333 / 335
MAK(8D)_XhoI<br>MAK(8R)_NheI	**AAGAAT***CTCGAG*CGCATTGTTTCCTAACTCTCAG<br>**AAGAAT***GCTAGC*TTTCTGCATTTCTCCCAATG	576	216 / 192
MERTK(9D)_NheI<br>MERTK(9D)_XhoI	**AAGAAT***GCTAGC*GGGTCTCACTCTGTTACCTAGC<br>**AAGAAT***CTCGAG*GTGTGACTGGCGTATTGTGC	889	249 / 271
PRCD (1D)_ XhoI<br>PRCD (1R)_ NheI	**AAGAAT***CTCGAG*TTAGAGTGGCAGCTCCTTGC<br>**AAGAAT***GCTAGC*TGGAGATGAGAGGCACAGC	979	203 / 599
PRPF8(4D)_ XhoI<br>PRPF8(4R)_ NheI	**AAGAAT***CTCGAG*TGGCCTGACAGACATGAGAC<br>**AAGAAT***GCTAGC*AAGGCCCACCTCAAGTAAGC	950	363 / 477
PRPF31(3 D)_XhoI<br>PRPF31(3 R)_NheI	**AAGAAT***CTCGAG*TAGCAAGGTGGCGGTCATAG<br>**AAGAAT***GCTAGC*GTCTGGGAAACCTCAAGCTG	835	221 / 429
RHO(1D)_XhoI<br>RHO(1R)_NheI	**AAGAAT***CTCGAG*GGACAGACAAGTCATGCAG<br>**AAGAAT***GCTAGC*GACAAGCGCATATTGCTCCA	945	249 / 240
RIMS1 (14D) _XhoI<br>RIMS1 (14R) _NheI	**AAGAAT***CTCGAG*GCGACACATCATTTAGAATGTACC<br>**AAGAAT***GCTAGC*TCCATGAATATGTCGCCTTG	647	224 / 251
RPGRIP1(7D)_XhoI<br>RPGRIP1(7R)_NheI	**AAGAAT***CTCGAG*GAAATGGTGTGAATGATTGCAG<br>**AAGAAT***GCTAGC*AATTTGCTCCAGCAATAGGC	497	205 / 268

Tails added at the beginning of the primer are indicated in bold. Enzyme restriction sites used in this study are indicated in italics.

Supplementary Table 3. Primers used for the site-directed mutagenesis.

Primer	Sequence (5’-3’)
PRCD(1D)	GCATGGCCAGGG**A**CCTG**T**G**A**AGGAAAAGGGTGGTGCACATGGC
PRCD(1R)	GCCATGTGCACCACCCTTTTCCT**T**C**A**CAG**GT**CCCTGGCCATGC

The nucleotides that have been modified are indicated in bold.

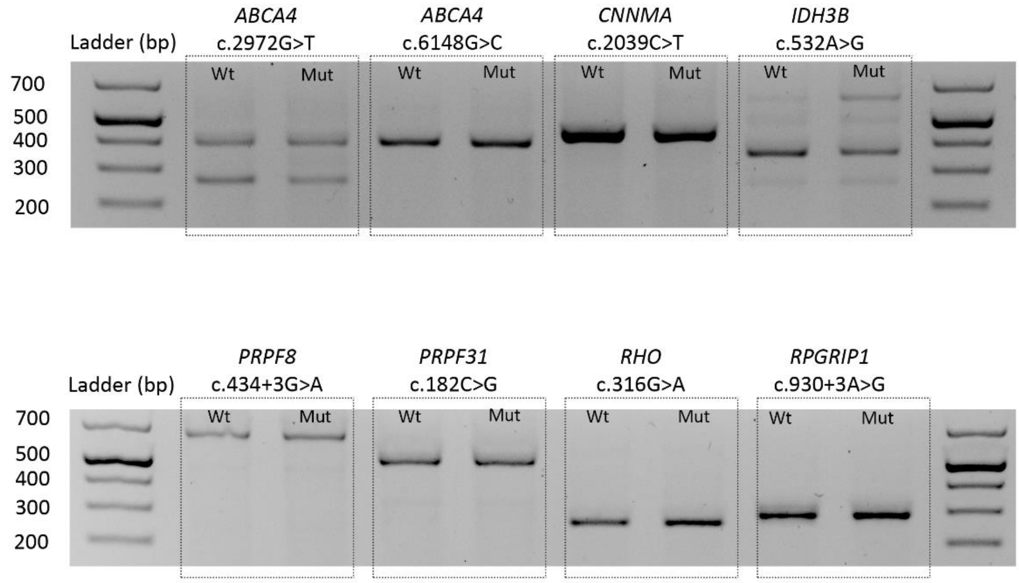

Supplementary Figure 1. Representation of the amplified product of the variants that did not affect the splicing.

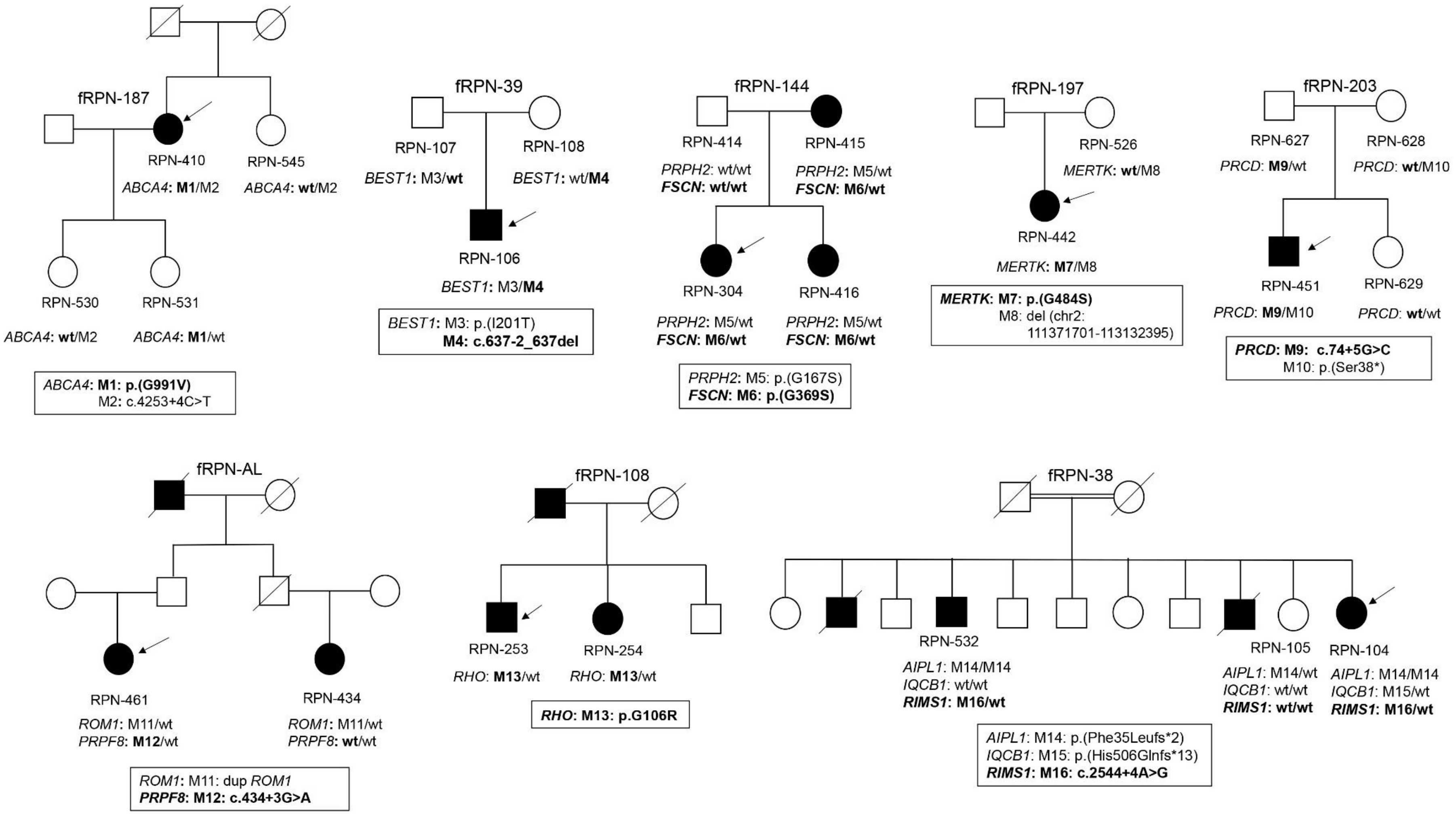

Supplementary Figure 2. Cosegregation analysis for the variants analysed in present study. The arrow indicates the index cases in each family. WT indicates "wild-type allele". M indicates the mutation analysed in each family. Individuals from whom DNA sample were available are indicated with identification numbers.

PRCD: wild-type Exon 1

AGACGGCAGTGGCTCCTGAGAGCTGGCTGGGGCCATTTTGGCCCCTCGCCTGTGGCCTT
CTGCAGACTTGGCCTGGGAGGGGATGGGGCAGCTGCGCCATGTGCACCACCCTTTTCCT
GC**T**CAG**CA**CCCTGGCCATGCTCTGGCGCCGCCGATTTGCCAACCGAGTCCAACCgtgag

c.74+5G>C

	Motif	HSF	MaxEnt
**Wildtype**	TTTCCT**G**C**T**CAG**CA**	88,43	7,39
**Mutated**	TTTCCT**T**C**A**CAG**GT**	96,30	12,6

Supplementary Figure 3. Modifications generated in the *PRCD* exon 1 sequence by site-directed mutagenesis to promote the recognition of the first exon.

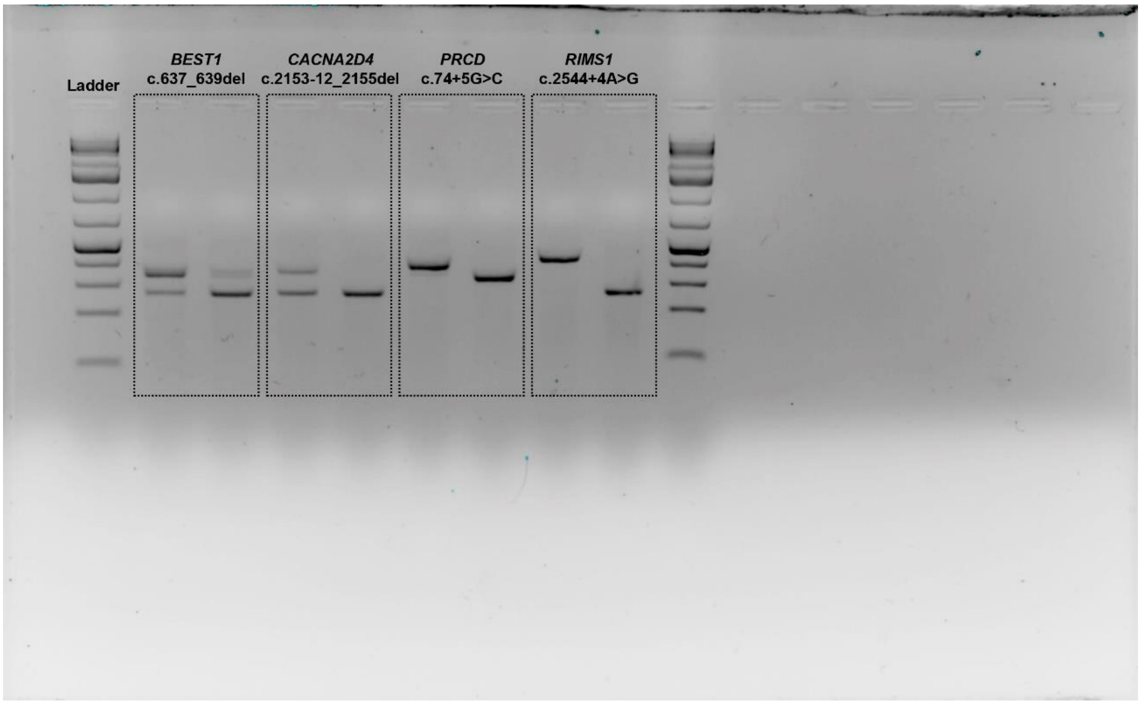

Raw Figure 1 (unprocessed version).

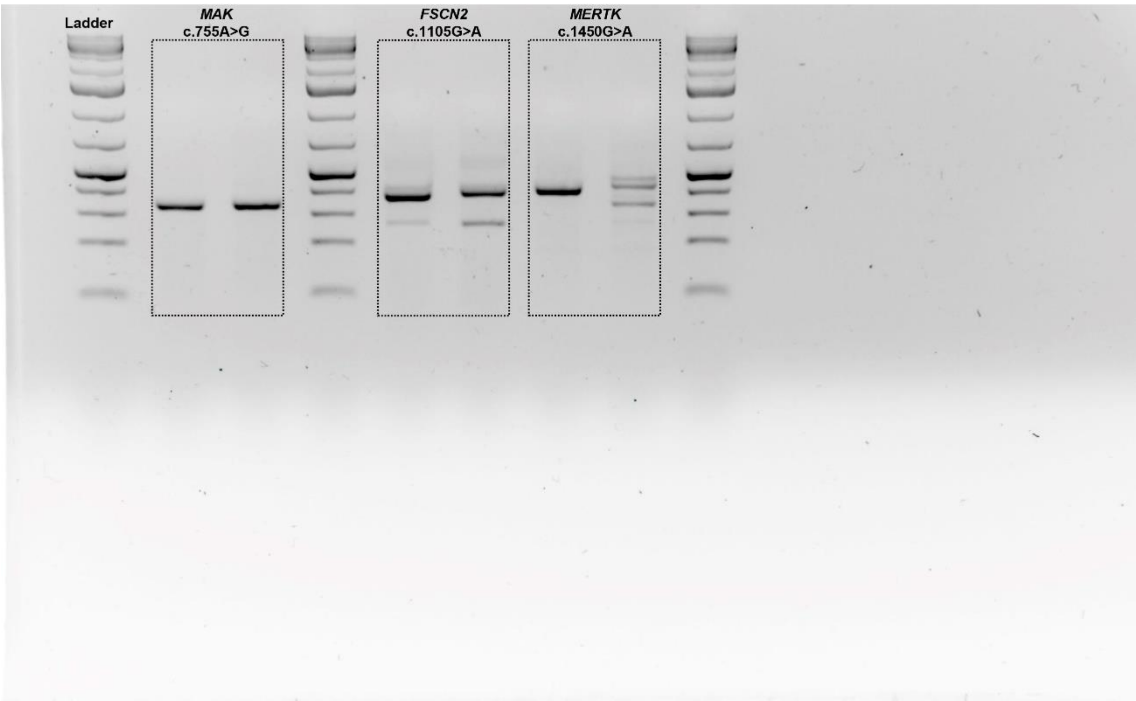

Raw Figure 1 (unprocessed version).

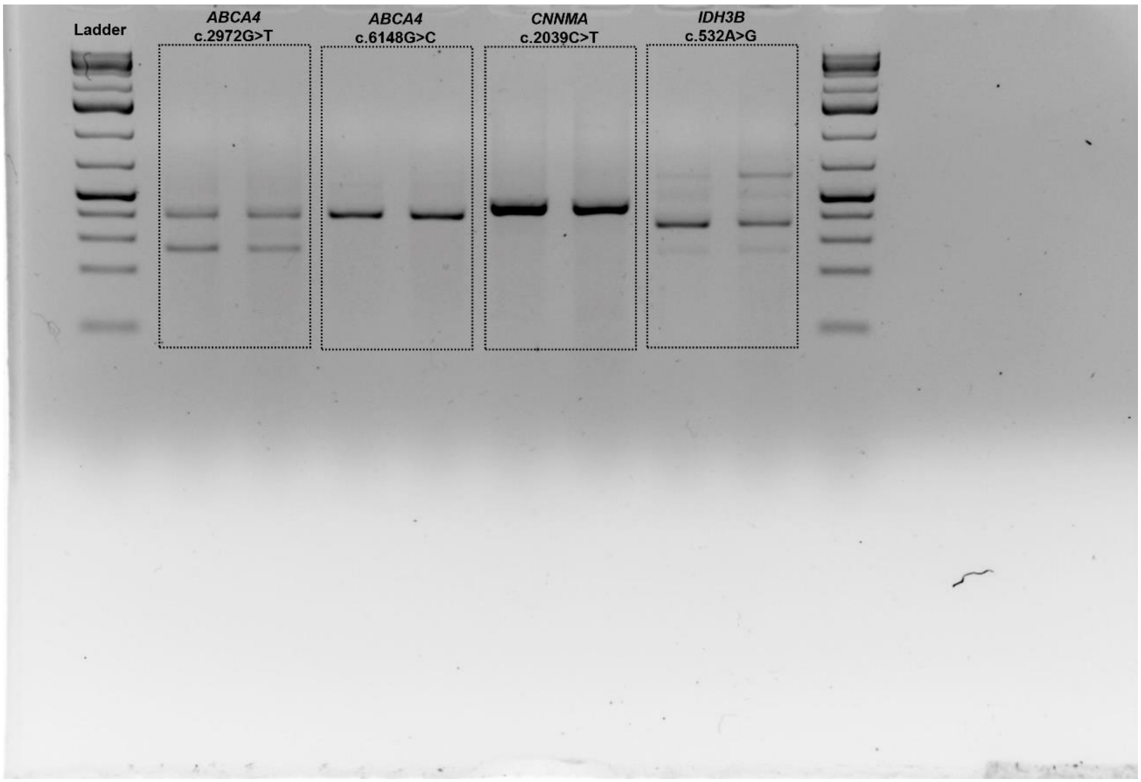

Raw Supplementary Figure 1 (unprocessed version).

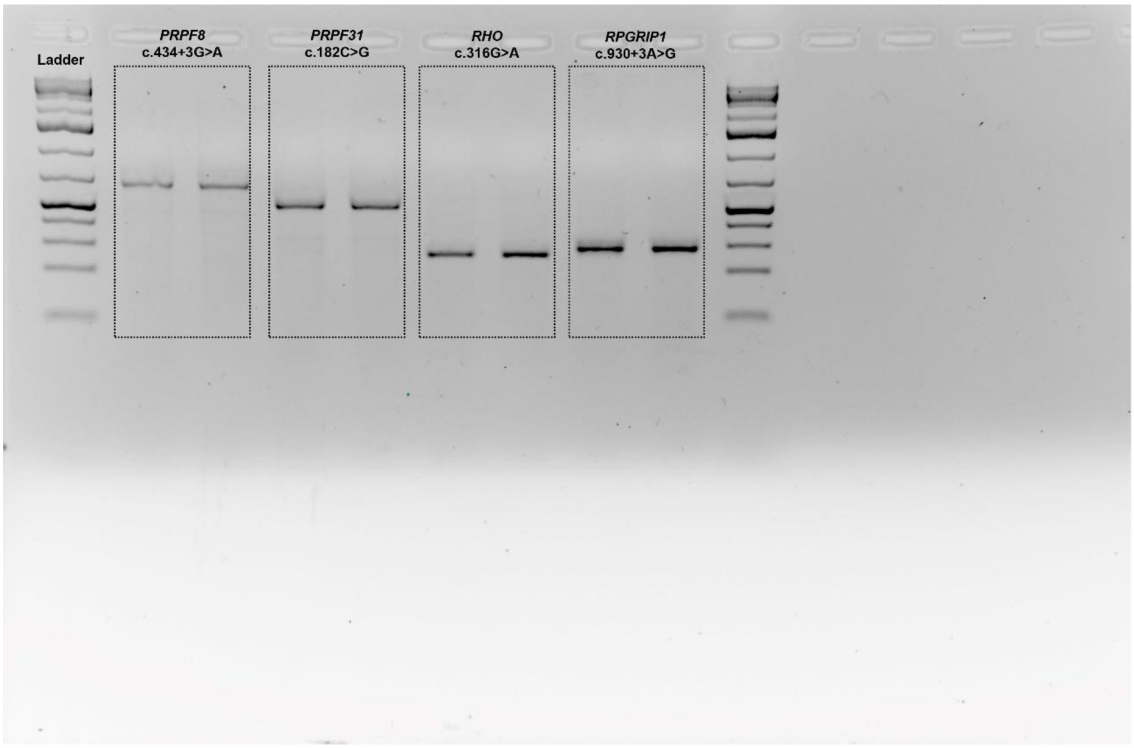

Raw Supplementary Figure 1 (unprocessed version).
